# Beyond Tissue Origin: Rethinking Experimental Model Selection in Rotator Cuff Research

**DOI:** 10.3390/medsci14040440

**Published:** 2026-07-27

**Authors:** Toru Ichiseki

**Affiliations:** 1 Department of Orthopaedic Surgery, Kanazawa Medical University, Daigaku 1-1, Uchinada-machi, Kahoku 920-0293, Ishikawa, Japan; tsy-ichi@kanazawa-med.ac.jp; 2 Division of Translational Research, Department of Life Science, Medical Research Institute, Kanazawa Medical University, Daigaku 1-1, Uchinada-machi, Kahoku 920-0293, Ishikawa, Japan

**Keywords:** rotator cuff, tenocytes, experimental model selection, cellular heterogeneity, tissue specificity, tendon biology, oxidative stress, translational research

## Abstract

Rotator cuff tears are among the most common tendon disorders and a major cause of shoulder pain and functional impairment. Increasing evidence indicates that rotator cuff pathology is a complex biological disease involving inflammation, oxidative stress, hypoxia, apoptosis, cellular senescence, and dysregulated extracellular matrix (ECM) remodeling. Consequently, in vitro studies using rotator cuff-derived cells have become important tools for investigating disease mechanisms and therapeutic targets. Although rotator cuff-derived cells are widely used and generally regarded as physiologically relevant experimental models, their use is limited by restricted tissue availability, inter-donor variability, and the predominance of specimens obtained from torn or degenerative tendons. Commercially available tenocytes offer advantages in accessibility, standardization, reproducibility, and scalability, yet their use in rotator cuff research remains limited because they are not derived from rotator cuff tissue. This Opinion article provides a multifaceted and balanced reappraisal of the view that rotator cuff-derived cells are indispensable for rotator cuff research and explores the potential role of commercially available tenocytes as complementary experimental models. Current cell-based models are discussed in light of the absence of established rotator cuff-specific molecular markers and the growing recognition of tendon cell heterogeneity. This article does not advocate replacing rotator cuff-derived cells. Rather, it proposes that commercially available tenocytes may serve as complementary experimental models for investigating biological processes—including inflammation, oxidative stress, ECM metabolism, and preliminary drug screening—for which tissue-specific biology may not always be required. A stepwise strategy involving screening in commercial tenocytes, validation in rotator cuff-derived cells, and confirmation in tissue, animal, or clinical studies is proposed to balance experimental accessibility with biological relevance. Such an approach may broaden research accessibility while facilitating translational research in rotator cuff disease.

## 1. Introduction

Rotator cuff tears are among the most prevalent tendon disorders and represent a major cause of shoulder pain and functional disability worldwide [1,2]. Epidemiological studies have demonstrated that the prevalence of rotator cuff tears increases with age, highlighting the growing clinical and socioeconomic burden associated with this condition [1,3]. Accordingly, elucidating the underlying disease mechanisms and developing effective therapeutic strategies remain important challenges in orthopaedic research and clinical practice [1,2].

Traditionally, rotator cuff tears were regarded primarily as mechanical injuries resulting from tendon overload and degeneration. However, accumulating evidence has revealed that rotator cuff pathology is a multifactorial biological process involving inflammation, hypoxia, oxidative stress, apoptosis, cellular senescence, and dysregulated extracellular matrix (ECM) remodeling [4,5,6,7,8,9,10,11,12,13]. These findings have shifted the focus of research toward understanding the molecular and cellular mechanisms that contribute to tendon degeneration and impaired healing. Consequently, cell culture models have become indispensable tools for investigating the biological pathways involved in rotator cuff disease [4,5,14,15,16].

Among the available experimental systems, human rotator cuff-derived cells isolated from surgical specimens are currently the most widely used cellular model [14]. Because these cells originate directly from diseased rotator cuff tissue, they are generally considered to provide a biologically relevant model for studies addressing tissue-specific disease mechanisms. As a result, they have become the preferred model for studying disease-associated cellular responses and evaluating potential therapeutic interventions.

Nevertheless, the use of rotator cuff-derived cells is associated with several practical and biological limitations. Tissue acquisition depends on surgical specimens, making cell isolation labor-intensive and often restricting sample availability. Moreover, patient-related factors such as age, tear size, diabetes mellitus, hyperlipidemia, and smoking status have been reported to influence tendon pathology [11,14,17,18,19,20,21,22]. These factors may also affect the biological characteristics of isolated cells and complicate the interpretation and reproducibility of experimental findings.

Commercially available tenocytes provide an alternative cell source that is readily accessible and may facilitate more standardized experimental workflows. Tenocyte-based experimental systems have also demonstrated biological utility in tendon research [23]. Despite these advantages, their use in rotator cuff research remains relatively limited because they are not derived from rotator cuff tissue. As a result, their applicability to studies focusing on tissue-specific aspects of rotator cuff biology may be considered limited.

However, the biological validity of any experimental model depends not only on the origin of the cells but also on how faithfully it reproduces the native tendon microenvironment, which is shaped by extracellular matrix composition, mechanical loading, and cell–matrix interactions [24]. Accordingly, experimental model selection should be guided not only by tissue origin but also by the ability of the experimental system to reproduce the biological processes of interest.

Despite the widespread use of rotator cuff-derived cells, criteria for selecting appropriate in vitro models remain incompletely defined and continue to be debated according to the biological question being addressed.

Against this background, this Opinion article does not advocate replacing rotator cuff-derived cells with commercially available tenocytes. Rather, it seeks to re-examine the view that rotator cuff-derived cells are indispensable for rotator cuff research. From this perspective, this Opinion article discusses the potential role of commercially available tenocytes as complementary experimental models and proposes a conceptual framework for experimental model selection in rotator cuff research.

## 2. Tissue Specificity and the Position of Rotator Cuff-Derived Cells as Experimental Models

The widespread use of rotator cuff-derived cells largely stems from the assumption that they retain tissue-specific biological characteristics and are therefore widely regarded as physiologically relevant in vitro models for studying rotator cuff disease. The rotator cuff is a specialized musculoskeletal structure that forms a tendon-to-bone attachment (enthesis) and is exposed to unique mechanical, biological, and age-related influences throughout life [12]. Consequently, rotator cuff-derived cells may exhibit biological properties that differ from those of tenocytes isolated from other anatomical locations.

However, the extent to which such tissue specificity can be defined and experimentally verified remains unclear. Moreover, the expression and functional role of Scleraxis are influenced by mechanical loading, illustrating that tendon-related cellular characteristics may also depend on the surrounding experimental environment [25]. Several markers, including Scleraxis (SCX), Tenomodulin (TNMD), Mohawk (MKX), Collagen Type I Alpha 1 (COL1A1), and Decorin (DCN), are widely used to characterize tendon cells and evaluate tenogenic differentiation [26,27,28]. While these markers are valuable indicators of tendon lineage, none are specific to the rotator cuff. Currently, no widely accepted molecular marker uniquely defining rotator cuff tenocytes has been established [26,27,28]. Therefore, although tissue origin may influence cellular behavior, molecular evidence directly supporting rotator cuff-specific cellular identity remains limited.

As a result, the term “rotator cuff-derived cells” currently refers primarily to cells isolated from rotator cuff tissue rather than to a molecularly defined cell population. Accordingly, tissue origin and molecular identity should not be regarded as synonymous concepts. Although such cells may indeed possess unique biological features, a universally accepted framework for defining rotator cuff-specific cellular identity is currently lacking.

Recent studies have revealed substantial cellular heterogeneity within tendon tissues through single-cell analyses and the identification of distinct tendon-resident cell populations [29,30,31]. In addition, tendon stem/progenitor cells (TSPCs) have been identified within tendon tissues and are thought to contribute to tissue homeostasis, repair, and regeneration [32,33,34]. More broadly, stem-cell-based regenerative approaches are increasingly being investigated in other musculoskeletal tissues, including the intervertebral disc [35]. This broader literature places tendon progenitor biology within the wider context of musculoskeletal regenerative research. The observation that progenitor cells derived from the tendon proper and peritenon possess distinct biological and tenogenic characteristics further supports the concept that tendon-derived cell populations are biologically heterogeneous [31]. These observations suggest that cell populations isolated from tendon specimens may contain not only mature tenocytes but also multiple progenitor-like subpopulations [30,31,32,33,34,35,36].

Importantly, no currently available isolation method can completely separate all cellular subtypes present within tendon tissue. Consequently, cultured tendon-derived cell populations should be regarded as inherently heterogeneous. Furthermore, phenotypic changes associated with monolayer culture and serial passaging may further alter cellular characteristics over time. Together, these factors may contribute to variability in experimental findings across studies and should therefore be considered when interpreting results obtained from tendon-derived cell cultures.

Taken together, these observations suggest that tissue origin alone may not be sufficient to define the biological validity of an experimental model. While rotator cuff-derived cells remain highly valuable research tools, their experimental relevance should be considered in the context of specific biological questions rather than being assumed solely on the basis of tissue provenance.

Notably, these considerations are not unique to rotator cuff-derived cells and also apply to commercially available tenocytes derived from other tendon tissues. Therefore, the value of a cellular model should be assessed not only according to its anatomical origin but also according to the biological question being addressed and the overall design of the experimental system.

## 3. Underappreciated Limitations of Rotator Cuff-Derived Cells

Rotator cuff-derived cells are widely used in vitro models for studying rotator cuff disease and are generally regarded as physiologically relevant experimental models. Nevertheless, several important limitations of this model type are often underappreciated.

First, substantial inter-donor variability exists among rotator cuff-derived cell populations. Because these cells are isolated from surgical specimens, their biological characteristics may be influenced by numerous patient-related factors, including age, sex, tear size, disease duration, diabetes mellitus, hyperlipidemia, and smoking status [11,14,17,18,19,20,21,22]. Such variability can complicate experimental reproducibility and make direct comparisons between studies challenging.

Second, most available human rotator cuff specimens are obtained from torn or degenerative tendons rather than from healthy tissue [14]. Torn rotator cuff tendons frequently exhibit inflammation, neovascularization, apoptosis, cellular senescence, fatty degeneration, and extensive extracellular matrix remodeling [5,6,7,8,9,10,11,12,13]. Therefore, cells isolated from these tissues may already possess disease-associated phenotypes before experimental manipulation. This consideration is particularly relevant because many in vitro studies expose rotator cuff-derived cells to inflammatory mediators such as interleukin-1β (IL-1β) or tumor necrosis factor-α (TNF-α) to model tendon degeneration. Although these approaches have provided important mechanistic insights, it remains unclear whether such experiments represent the induction of pathology in otherwise healthy tendon cells or the amplification of pathology in cells that are already biologically altered. Thus, commonly used rotator cuff-derived cell cultures may be more accurately viewed as patient-derived pathological models rather than models of normal tendon biology.

Third, tendon cells are known to undergo phenotypic drift during in vitro expansion. Previous studies have demonstrated that serial passaging is associated with reduced expression of tendon-related genes and progressive divergence from the native cellular phenotype [37]. Moreover, culture conditions themselves—including serum concentration, substrate composition, and cell density—can significantly influence cellular behavior and gene expression profiles [38,39,40]. Consequently, cultured rotator cuff-derived cells should not be assumed to fully recapitulate their in vivo counterparts.

Furthermore, recent single-cell analyses have highlighted the remarkable cellular complexity of tendon tissues [29,30,34]. Conventional two-dimensional monolayer cultures capture only a subset of this cellular diversity and therefore fail to recapitulate many essential features of the native rotator cuff microenvironment. In vivo, tendon tissue contains multiple interacting cell populations, including tenocytes, fibrochondrocytes, osteoblast-lineage cells, vascular cells, and immune cells [29,30,31,34]. Mechanical loading, extracellular matrix architecture, oxygen gradients, cell–cell interactions, and cell–matrix interactions also contribute substantially to tissue homeostasis and disease progression [39,41,42]. Accordingly, findings obtained from cultured cells should be interpreted within the context of these inherent limitations.

Therefore, although rotator cuff-derived cells remain valuable experimental tools, it is important to recognize that currently available culture systems do not fully replicate the biological complexity of the native rotator cuff. Appreciating these limitations may facilitate a more balanced evaluation of alternative experimental models and encourage the development of complementary research strategies.

## 4. The Rationale for Utilizing Commercial Tenocytes

One of the principal advantages of commercially available tenocytes is their accessibility and experimental standardization.

Studies utilizing rotator cuff-derived cells are inherently dependent on the availability of surgical specimens. Consequently, research productivity may be influenced by patient recruitment, surgical volume, and institutional resources. In some settings, limited access to rotator cuff tissue represents a substantial barrier to conducting basic tendon research.

Commercially available tenocytes can be obtained when needed and may reduce variability associated with tissue procurement and initial cell isolation. Such standardization may improve experimental reproducibility and facilitate more consistent comparisons across studies [43]. Given the growing emphasis on reproducibility in biomedical research, these advantages warrant careful consideration.

Importantly, the availability of commercial tenocytes may broaden participation in tendon biology research. Access to human rotator cuff tissue is restricted to a relatively small number of institutions, whereas commercially available cells can be utilized by investigators without direct access to surgical specimens. Lowering such logistical barriers may encourage broader participation and foster innovation within the field.

Evidence from tendon biology further suggests that many fundamental cellular processes are shared across different tendon types. Studies investigating tendon mechanobiology, tissue engineering, extracellular matrix remodeling, and oxidative stress have frequently employed tenocytes derived from non-rotator cuff tendons and have generated important biological insights [15,16,39,42].

This observation is particularly relevant because many experimental paradigms commonly used in rotator cuff research do not necessarily address rotator cuff-specific biology. Models involving inflammatory stimulation, oxidative stress, hypoxia, apoptosis, or extracellular matrix remodeling are often designed to investigate cellular responses that may be conserved across tendon tissues [4,5,6,7,8,9,15,16].

For example, stimulation with IL-1β or TNF-α induces matrix metalloproteinase expression, inflammatory cytokine production, and alterations in tendon-related gene expression in rotator cuff-derived cells [5,6,13,44]. Similar inflammatory and stress-related responses have also been reported in tenocytes derived from other anatomical locations [15,16,44]. Likewise, oxidative stress-induced apoptosis and extracellular matrix dysregulation have been observed across multiple tendon cell models [15,16].

These observations do not imply that commercially available tenocytes are biologically equivalent to rotator cuff-derived cells. Rather, they suggest that commercial tenocytes may serve as useful experimental platforms for investigating fundamental biological processes, including inflammation, oxidative stress, apoptosis, extracellular matrix metabolism, and preliminary drug screening. Commercial tenocytes may therefore be particularly useful for preliminary mechanistic screening, provided that key findings are subsequently validated in rotator cuff-derived cells, tissue-level models, or other physiologically relevant systems. More broadly, the biological utility of tenocyte-derived products is supported by in vivo evidence showing that tenocyte-derived conditioned medium promotes histological and functional tendon recovery [23].

At the same time, important limitations must be acknowledged. Most commercially available tenocytes are derived from tendons other than the rotator cuff, and the extent to which their biological characteristics overlap with those of rotator cuff-derived cells remains incompletely understood. Direct comparative studies between these cell populations remain limited.

Nevertheless, given the absence of established rotator cuff-specific molecular markers and the shared expression of commonly used tendon-related markers such as SCX, TNMD, MKX, and COL1A1 [26,27,28], it may be premature to dismiss the potential research value of commercially available tenocytes solely on the basis of tissue origin.

Accordingly, commercially available tenocytes should be viewed as complementary models rather than replacements for rotator cuff-derived cells, and their broader application will require validation through future direct comparative studies. A comparison of the major strengths and limitations of both cell sources is summarized in Table 1.

## 5. The Contribution of Experimental System Design to Model Validity

The native rotator cuff environment is not composed of a single cell type. Rather, it consists of a complex network of tenocytes, fibrochondrocytes, osteoblast-lineage cells, vascular cells, and immune cells that interact to maintain tissue homeostasis and coordinate responses to injury [29,30,34].

In addition, hypoxia has been implicated in the pathogenesis of rotator cuff degeneration [12], while mechanical loading is recognized as a critical regulator of tendon homeostasis, adaptation, and repair [24,25,45,46]. These biological factors operate simultaneously within a highly organized extracellular matrix and contribute substantially to tendon physiology and pathology.

However, most currently used in vitro models—including cultures of rotator cuff-derived cells—do not adequately reproduce these features. Conventional monolayer culture systems largely eliminate cell–cell interactions, extracellular matrix architecture, physiological oxygen gradients, and mechanical loading conditions that are present in vivo [39,41].

Recent advances in tissue engineering have enabled the development of more sophisticated experimental systems, including three-dimensional culture platforms, mechanically stimulated culture systems, hypoxic culture conditions, and extracellular matrix-based scaffolds [39,41,42,45]. These approaches aim to recreate aspects of the native tendon microenvironment that are absent in traditional cell culture models [24,41].

From this perspective, the biological relevance of an experimental model may depend not only on the anatomical origin of the cells but also on the extent to which the experimental system reproduces key features of the native tissue environment [24,45]. This does not imply that cell origin is unimportant. Rather, it suggests that tissue origin represents only one component of model validity.

Importantly, no single experimental model can fully reproduce the complexity of rotator cuff disease. Therefore, selecting the most appropriate model should depend on the biological question being addressed. In some circumstances, factors such as culture conditions, mechanical stimulation, oxygen tension, extracellular matrix composition, and multicellular interactions may influence experimental outcomes substantially and may, in some contexts, be as important as cell origin [24,39,41,42,45,47].

**Figure 1:**
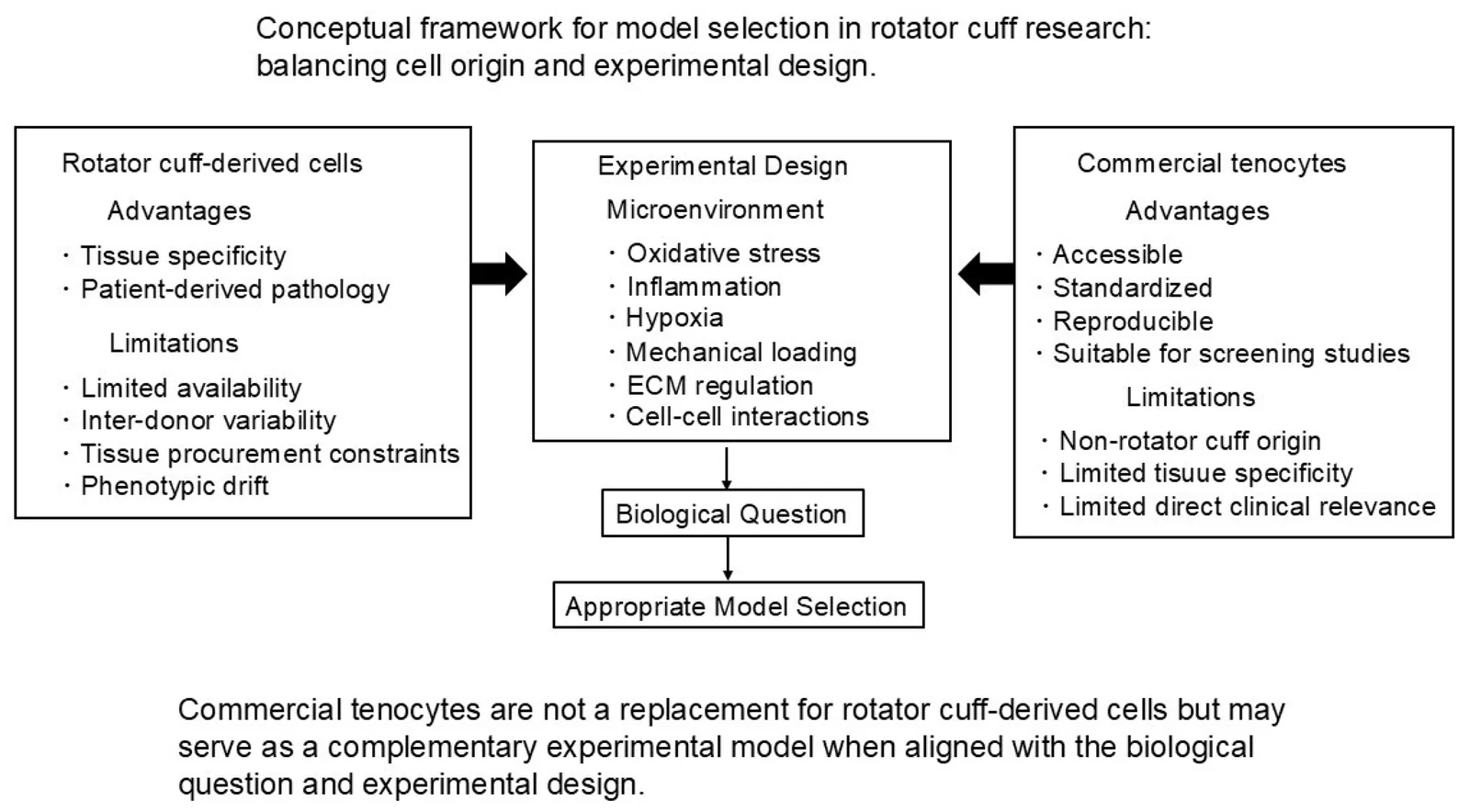
Conceptual framework for experimental model selection in rotator cuff research.

Accordingly, experimental model selection may benefit from moving beyond a binary distinction between rotator cuff-derived and non-rotator cuff-derived cells and instead focusing on how effectively a given system reproduces the biological phenomenon of interest. To facilitate this perspective, we propose the following conceptual framework to assist investigators in selecting experimental models according to biological objectives rather than tissue origin alone. The proposed framework is summarized in Figure 1.

The schematic integrates cell source with key features of the native tendon microenvironment—including extracellular matrix composition, mechanical loading, oxygen tension, and multicellular interactions—to guide experimental model selection according to the research objective.

## 6. When Should Commercial Tenocytes Be Used—And When Should They Not?

The preceding discussion suggests that commercially available tenocytes are unlikely to be equally suitable for all research questions. Their utility depends largely on the biological process under investigation.

Commercial tenocytes may be particularly useful for studies focusing on fundamental cellular mechanisms that are shared across tendon tissues. Examples include investigations of:

- Oxidative stress responses;
- Inflammatory signaling pathways;
- Apoptosis;
- Autophagy;
- Mitochondrial dysfunction;
- Extracellular matrix metabolism;
- Drug screening;
- Biomaterial evaluation;
- Intracellular signaling mechanisms.

In these contexts, tendon-wide biological processes may be of greater importance than strict tissue specificity [4,5,6,7,8,9,13,15,16,44,46].

Similarly, commercially available tenocytes may be advantageous for hypothesis generation, mechanistic studies, and early-stage screening because their accessibility and relative standardization facilitate reproducible exploratory research.

Conversely, certain research questions require models that preserve tissue-specific or patient-specific characteristics. Examples include:

- Patient-specific disease modeling;
- Rotator cuff-specific transcriptomic analyses;
- Precision medicine approaches;
- Correlation studies involving clinical outcomes;
- Enthesis-specific biology;
- Investigations of rotator cuff-specific biomechanical adaptation.

In these situations, rotator cuff-derived cells, tissue-level analyses, or patient-derived samples are likely to remain indispensable [8,10,12,29,30]. These distinctions are summarized in the research-objective-based framework presented in Table 2.

## 7. Future Perspectives

One promising direction for future rotator cuff research is the development of strategies that enhance the physiological relevance of commercially available tenocyte models.

Rotator cuff degeneration is characterized by a complex interplay of inflammation, hypoxia, oxidative stress, aging-related changes, and extracellular matrix remodeling [4,5,6,7,8,9,12,13]. Accordingly, exposing commercial tenocytes to inflammatory cytokines, oxidative stress, hypoxic conditions, or mechanical stimulation may allow investigators to reproduce selected aspects of disease-related microenvironments [4,7,12,13,45,46].

Among these factors, oxidative stress has emerged as a particularly important contributor to tendon degeneration, apoptosis, and extracellular matrix dysregulation [4,5,7,8,9,15,16,48]. Oxidative stress-related signaling pathways and oxidative stress-induced cellular injury have been observed not only in rotator cuff-derived cells but also in tenocytes from other anatomical locations [4,5,7,8,9,15,16]. More broadly, human studies have demonstrated that systemic redox status is measurable and responsive to physiological stress, including variations in exercise intensity [49]. More specifically, altered oxidative status has been reported in patients with rotator cuff tears and tendinopathy, supporting the clinical relevance of oxidative stress in vivo [9,48]. While such observations do not establish biological equivalence between different tendon cell populations, they suggest that commercially available tenocytes may represent useful platforms for investigating oxidative stress-related mechanisms and identifying potential therapeutic targets.

Future progress may also depend on the development of multicellular experimental systems. The rotator cuff enthesis consists of multiple interacting cell populations, including tenocytes, fibrochondrocytes, osteoblast-lineage cells, synovial cells, vascular cells, and immune cells [29,30,34]. Consequently, understanding disease mechanisms through single-cell-type cultures alone is inherently challenging.

Importantly, the objective is not simply to create co-culture systems but to incorporate biologically relevant cellular interactions into experimental design. Combining multiple cell populations with three-dimensional culture platforms, extracellular matrix engineering, and controlled mechanical stimulation may allow more faithful reproduction of native tissue biology [24,25,39,41,42].

At the same time, advances in single-cell RNA sequencing and spatial transcriptomics continue to reveal previously unrecognized cellular diversity within tendon tissues [29,30,34]. These technologies are likely to provide important insights into disease-associated cellular interactions and may ultimately help define more biologically relevant experimental models.

Clinical systematic reviews in other tendon disorders, including Achilles tendinopathy, further illustrate the importance of downstream clinical evaluation when translating mechanistic findings into therapeutic strategies [50]. Naturally, comprehensive understanding of rotator cuff disease and validation of therapeutic interventions will continue to require animal models and clinical studies [51]. Nevertheless, in vitro tendon cell models remain essential tools for identifying candidate pathways, exploring molecular mechanisms, and conducting early-stage therapeutic screening.

In this context, a stepwise research strategy may be considered, consisting of:

(1) Initial screening using commercially available tenocytes;
(2) Validation using rotator cuff-derived cells;
(3) Confirmation through animal models and clinical investigations.

This proposed framework should be regarded as a hypothesis-generating conceptual model rather than an evidence-based recommendation. Even so, it may provide practical guidance for selecting experimental models according to specific research objectives in future studies.

Such a framework may help balance research efficiency with biological relevance while facilitating translational progress.

Ultimately, future evaluation of experimental models may shift from a primary focus on tissue origin toward a broader assessment of how effectively a model reproduces relevant biological microenvironments [45,47]. Within such a framework, commercially available tenocytes may assume a valuable role as complementary experimental resources in rotator cuff research.

Equally important, direct comparative studies between commercially available tenocytes and rotator cuff-derived cells represent a critical future research priority. Such investigations may help define the biological overlap and distinctions between these models and establish clearer guidance regarding their appropriate applications.

## 8. Conclusions

This Opinion article reconsidered the role of commercially available tenocytes in rotator cuff research.

Rotator cuff-derived cells remain valuable experimental models for investigating disease-specific biological processes and continue to provide important insights into tendon pathology. However, their use is accompanied by several limitations, including inter-donor variability, restricted access to healthy control tissue, phenotypic drift during culture, and the inability of current in vitro systems to fully reproduce the native tissue microenvironment.

Commercially available tenocytes, although not derived from the rotator cuff, offer distinct advantages in accessibility, standardization, reproducibility, and scalability. Moreover, no universally accepted molecular marker currently exists that uniquely defines rotator cuff tenocytes, and many commonly used tendon-related markers are shared across tendon tissues.

Therefore, the value of an experimental model should not be judged solely on the basis of tissue origin. Rather, experimental validity depends on how appropriately a model addresses the biological question being investigated and how effectively the experimental system reproduces relevant aspects of the native microenvironment.

This article does not advocate replacing rotator cuff-derived cells with commercially available tenocytes. Instead, commercial tenocytes may serve as useful complementary models for investigating fundamental biological processes, including oxidative stress, inflammatory responses, extracellular matrix metabolism, and preliminary therapeutic screening.

Future advances in preconditioning strategies, three-dimensional culture systems, mechanobiological platforms, and multicellular models may further enhance the physiological relevance of commercially available tenocyte-based systems. At the same time, comparative studies between commercial tenocytes and rotator cuff-derived cells will be essential for defining the strengths, limitations, and optimal applications of each model.

Ultimately, progress may depend not only on which experimental model is selected, but also on which biological phenomena are being modeled. From this perspective, commercially available tenocytes deserve further systematic evaluation as complementary experimental resources capable of supporting future advances in tendon biology and translational rotator cuff research.

## Figures and Tables

**Table 1:** Comparison of rotator cuff-derived cells and commercially available tenocytes as complementary experimental models.

Characteristics	Rotator Cuff-Derived Cells	Commercially Available Tenocytes
Tissue origin	Rotator cuff tendon	Various tendon tissues
Tissue specificity	High	Less defined
Patient-derived pathology	Present	Generally absent
Accessibility	Limited	High
Availability of healthy controls	Limited	More feasible
Standardization	Moderate	High
Reproducibility	Variable	Relatively high
Inter-donor variability	High	Lower
Scalability	Limited	High
Suitability for mechanistic studies	High	High
Suitability for screening studies	Moderate	High
Suitability for patient-specific studies	High	Low
Clinical relevance	High	Moderate

Notes. Rotator cuff-derived cells provide tissue-specific and patient-derived biological information, making them particularly valuable for studies investigating rotator cuff pathology. However, their use is limited by restricted tissue availability, substantial inter-donor variability, limited access to healthy control tissue, and challenges related to scalability and standardization. In contrast, commercially available tenocytes offer greater accessibility, standardization, reproducibility, and scalability, making them attractive experimental platforms for mechanistic investigations and high-throughput screening applications. Both cell sources are subject to phenotypic drift during in vitro culture and do not fully reproduce the native tendon microenvironment. Therefore, the comparison presented here should not be interpreted as a hierarchy between experimental models but rather as a conceptual framework highlighting their complementary strengths and limitations. The suitability of each model ultimately depends on the biological question being addressed and the overall design of the experimental system.

**Table 2:** Proposed framework for experimental model selection according to research objectives in rotator cuff research.

Research Objective	Commercial Tenocytes	Rotator Cuff-Derived Cells
Oxidative stress mechanisms	++	++
Inflammatory signaling	++	++
Apoptosis	++	++
Autophagy	++	++
Mitochondrial dysfunction	++	++
ECM metabolism and remodeling	++	++
Drug screening	++	+
Biomaterial evaluation	++	+
Intracellular signaling analysis	++	++
General tendon mechanobiology	++	++
Tendon tissue engineering	++	++
Patient-specific pathology	−	++
Rotator cuff-specific transcriptomics	−	++
Clinical correlation studies	−	++
Precision medicine approaches	−	++
Enthesis biology	±	++
Rotator cuff-specific biomechanics	±	++

**Notes.** This hypothesis-generating framework summarizes the relative suitability of each cell source for different research objectives and should not be interpreted as a validated guideline or a hierarchy of biological value. Model selection should consider the biological question, the degree of tissue specificity required, and the ability of the experimental system to reproduce the phenomenon of interest. Direct comparative studies are needed to refine these classifications. **Symbol definitions:** ++ = Highly suitable; + = Suitable; ± = Limited suitability; − = Not recommended. **Abbreviations:** ECM, extracellular matrix.

## Data Availability

No new data were created or analyzed in this study. Data sharing is not applicable.
